# Preparing Emergency Medical Services for Prehospital Buprenorphine: Examining a Train-the-Trainer Approach to Strengthening the Overdose Chain of Survival

**DOI:** 10.1016/j.acepjo.2026.100452

**Published:** 2026-07-01

**Authors:** Maria E. Paschke, Saad T. Siddiqui, Joshua Wilson, Gerard Carroll, Melissa Kroll, Bradley Ray, Rachel P. Winograd

**Affiliations:** 1 Missouri Institute of Mental Health, University of Missouri–St. Louis, St. Louis, Missouri, USA; 2 Department of Emergency Medicine, Cooper University Hospital, Cooper Medical School at Rowan University, Camden, New Jersey, USA; 3 Washington University School of Medicine, St. Louis, Missouri, USA; 4 Research Triangle Institute (RTI) International, Durham, North Carolina, USA; 5 Psychological Sciences, University of Missouri–St. Louis, St. Louis, Missouri, USA

**Keywords:** emergency medical services, buprenorphine, opiate overdose, substance-related disorders, implementation science, program development, capacity building

## Abstract

**Objectives:**

Opioid-related overdoses remain the leading cause of accidental death in the US. Many jurisdictions are exploring emergency medical services (EMS)-initiated prehospital buprenorphine induction at overdose scenes to reduce subsequent risk. This study evaluated a training strategy preparing EMS agencies to implement these protocols by examining barriers and facilitators affecting their readiness to integrate buprenorphine into standard care.

**Methods:**

Leadership from 6 Missouri EMS agencies participated in a 6-month training program to support prehospital buprenorphine implementation. We described this evidence-informed frame-and-foster training approach and present findings from a readiness checklist and interviews with 8 trainees. Analysis followed a deductive, exploration, preparation, implementation, sustainment (EPIS)-guided framework approach to assess trainees’ perceptions of the training and inner and outer barriers and facilitators to agency-wide implementation.

**Results:**

All agencies reported readiness, with (1) frontline staff training and (2) partnering with peer-linkage programs as areas for further preparation. Training increased confidence and clarified workflows. Remaining inner barriers within EMS agencies included stigma among paramedics toward people who use drugs, staff resistance, and burnout, while facilitators included clear protocols and integration into community paramedicine programs. Outer barriers included inconsistent community buprenorphine availability, while facilitators included continued training and state support.

**Conclusions:**

This evaluation provides early insights into how a structured training strategy can prepare EMS agencies to implement prehospital buprenorphine. Findings can inform future policy and practice to ensure EMS can effectively incorporate buprenorphine induction into overdose response and improve patient outcomes.

**The Bottom Line**

What do emergency medical services (EMS) agencies need to be prepared to implement prehospital buprenorphine protocols for opioid overdose patients? A 6-month training program with 6 Missouri EMS agencies framed buprenorphine within the “Overdose Chain of Survival” and fostered peer-to-peer learning with practice experts and peer treatment-linkage programs. Training improved confidence and workflows, with all 6 agencies reporting readiness to launch. Remaining barriers, such as stigma, staff burnout, and treatment access, highlight targets for future efforts.

## 1 Introduction

### 1.1 Background

More than 1 million people have died from opioid overdoses in the US over the past 2 decades.^1^ Research suggests buprenorphine, a medication for opioid use disorder (MOUD), is effective at reducing overdose deaths.^2^^,^^3^ Federal, state, and local efforts to address high rates of opioid-related overdose have focused on expanding access to buprenorphine,^4^, ^5^, ^6^, ^7^ yet this medication remains underutilized.^8^^,^^9^ In response, there have been targeted efforts to implement buprenorphine at overdose touchpoints,^10^, ^11^, ^12^, ^13^ including emergency medical services (EMS).^14^

### 1.2 Importance

Buprenorphine alleviates opioid withdrawal symptoms through partial agonism at the μ-opioid receptor without producing the level of euphoria or respiratory depression as full agonist opioids.^15^ Buprenorphine, even when diverted for nonmedical prescription drug use, is associated with a reduced risk of fatal overdose.^16^^,^^17^

Buprenorphine is well-suited for use in an EMS context given its rapid onset, minimal need for titration, and low risk of respiratory depression.^18^^,^^19^ In June 2019, the New Jersey State Health Commissioner released an executive order allowing buprenorphine administration by paramedics.^20^ Initial programs in New Jersey adapted buprenorphine induction protocols for the field, and several studies have demonstrated the feasibility and safety of this intervention.^21^, ^22^, ^23^ Following the federal Medication Access and Training Expansion Act in December 2022, EMS induction protocols have rapidly expanded.^24^ A 2025 review of prehospital buprenorphine protocols identified ten studies reporting outcomes, with most documenting high rates of initiation, linkage to follow-up care, and patient retention.^25^

However, implementing buprenorphine induction protocols remains novel. Wampler et al^26^ (2025) found nearly half of EMS clinicians opposed implementing buprenorphine protocols, citing both implementation-related concerns (eg, lack of confidence in administration) and attitudinal concerns (eg, patient risk compensation). Early evaluation demonstrated these barriers appear in practice, including negative EMS attitudes,^27^ inconsistent implementation,^28^ patient engagement difficulty,^28^ and access to follow-up care.^27^^,^^28^ Studies cited a lack of training as contributing to these challenges.^27^^,^^28^ Different administration models may require tailored training. Treatment with buprenorphine was limited to community paramedicine programs in 19% of protocols.^25^ Community paramedics are specially trained EMS who provide nonemergency care to address health care gaps,^29^ which may be particularly useful for substance use services.

### 1.3 Goals of This Investigation

This investigation assessed readiness, barriers, and facilitators to implementing prehospital buprenorphine immediately following a structured training program and prior to formal implementation. This training approach framed paramedic initiation of buprenorphine within an "Overdose Chain of Survival" (Supplementary Appendix 1), adapted from the Cardiac Chain of Survival,^30^^(p12)^ to help paramedics improve outcomes across overdose response, prehospital buprenorphine administration, and linkage facilitation^31^^,^^32^ (a similar model was proposed by Dernback et al^33^). Findings from readiness surveys and trainee interviews inform efforts to scale similar protocols.

## 2 Methods

### 2.1 Program Design and Implementation

This study evaluated a new training strategy supporting prehospital buprenorphine implementation across 6 Missouri EMS agencies. In 2023, the Missouri Department of Mental Health provided funding to the University of Missouri-St. Louis and the Missouri Institute of Mental Health, and 6 EMS agencies were selected for an initial pilot (Table 1) to provide prehospital buprenorphine to patients presenting with an opioid-associated overdose, regardless of transport destination. To prepare for implementation, the team developed a frame-and-foster training strategy. This approach was informed by prior projects where similar training strategies supported successful implementation outcomes.^34^^,^^35^ The framing component used targeted terminologies and shared goals to align healthcare concepts with broader overdose prevention strategies. Central to this was the “overdose chain of survival” framing, a framework that situates overdose response along a continuum and clarifies their role within it. The fostering strategy entailed colearning and codevelopment with practitioners, practice experts, and partners across the continuum of care.

**Table 1:** Pilot emergency medical services (EMS) agencies: department characteristics and community context.

County	Department type	Community paramedics program	Department Size	Coverage area	Annual call volume (approximate)	Approximate annual naloxone administrations	Crude fatal overdose rate (per 100,000)∗
Jackson [J1]	Fire protection	Yes	150	60 sq mi	10,000	1200	22.9
Jackson [J2]	Fire protection	Yes	50	10 sq mi	8000	144	22.9
Boone [B]	Hospital-based ambulance service	No	113	686 sq mi	20,000	144	19.9
Jefferson [J3]	Ambulance service	No	42	181 sq mi	10,000	180	24.5
St. Louis County [S1]	Fire protection	Yes	140	54 sq mi	17,000	600	26.7
St. Louis County [S2]	Hospital-based ambulance service	No	134	110 sq mi	34,000	3100	26.7

County is based on where the EMS agency was located; call volume and naloxone administration are based on yearly estimates from the agencies. Fatal overdose rates were calculated using the 2023 opioid overdose fatalities data from the Missouri Department of Health and Senior Services, and 2023 American Community Survey 5-year Estimates.

Training occurred through monthly 2-hour community-of-practice meetings between September 2023 and February 2024. Participants included subject matter experts from New Jersey who pioneered paramedic buprenorphine induction, representatives from Missouri treatment-linkage programs, and leadership from the 6 EMS agencies, including Medical Directors and Community Paramedics selected by their agencies (hereafter referred to as “trainees”).

Training provided didactic information about opioid use disorder and buprenorphine induction protocols and allowed for discursive learning on barriers to implementation, like electronic health record adaptation, medication procurement, and controlled substance storage and transport. The training used a train-the-trainer model, where initial trainees who participated in the 6-month frame-and-foster training were prepared to train agency frontline staff. They were also provided with challenge coins (Supplementary Appendix 2) to honor paramedics initiating prehospital buprenorphine in the field.

### 2.2 Setting and Participants

In this study, we examined readiness checklist survey results and interview transcripts from key informants representing 6 pilot EMS agencies. Eligible participants were trainees who attended community-of-practice meetings and received frame-and-foster training; participation was voluntary. Data collection included 4 individual interviews and 2 group interviews, each with 2 trainees. Data saturation was reached after interviews with at least 1 representative from each participating agency produced sufficiently overlapping information.

### 2.3 Data Collection

Interviews were conducted in February 2024 upon training completion by authors MP (a White female research scientist) and JW (a Black male project coordinator and former paramedic), members of the university team selected for complementary evaluation and operational expertise. Both interviewers had prior relationships with trainees through community-of-practice meetings. Interviews were framed as quality improvement to identify technical assistance needs and inform EMS practice. Methods are reported in accordance with Consolidated Criteria for Reporting Qualitative Research (COREQ) guidelines to ensure reflexivity and transparency.

The project team developed a 10-item readiness checklist outlining the key requirements for EMS agencies to assess successful preparation for beginning implementation (Table 2). Items were informed by core topics from the community-of-practice meetings and common implementation literature domains. Interviews followed a semistructured interview guide and lasted approximately 45 minutes over Zoom. The guide walked participants through the checklist, with trainees responding verbally (Supplementary Appendix 3: Readiness Checklist and Interview Guide). The interviewers took field notes, and all data were recorded and transcribed verbatim.

**Table 2:** Readiness for prehospital buprenorphine implementation among pilot emergency medical services (EMS) agencies posttraining: readiness checklist results (n = 6).

Checklist item	No. of districts reporting “yes”
Training and staffing (frontline context)	
1 Has a department representative engaged in at least 4 h of communities-of-practice training to learn best practices for implementing Bupe Rescue Protocol?	6
2 Does your department have a plan for how they will train your providers on buprenorphine rescue best practices and expectations?	4
3 Does your department have enough paramedics and vehicles to implement this pilot?	6
Buprenorphine first protocol (fidelity)	
4 Is your department prepared to use the COWS score for evaluating patients postoverdose?	6
5 Is your department prepared to make referrals to Missouri’s EPICC program or other service linkage programs?	5
6 Is your department prepared to provide leave-behind naloxone kits following overdose responses?	5
7 Does your department have the capacity to track and share administrative data (with appropriate privacy protocols) related to the prehospital buprenorphine (such as calls for service)	6
Capacity and context (administrative)	
8 Is your medical director willing to support your department’s ongoing participation?	5
9 Buprenorphine is a Schedule III substance. Does your agency have the required state and federal controlled substances registrations?	6
10 Does your department have enough financial capacity to purchase medication and other supplies to successfully implement this pilot?	6

COWS, clinical opiate withdrawal scale; EPICC, Missouri’s Engaging Patients in Care Coordination.

### 2.4 Framework Application

The exploration, preparation, implementation, sustainment (EPIS) framework guided the evaluation. The EPIS framework has been widely used to understand how interventions are introduced and maintained, particularly in healthcare and human services.^36^ As a “process model,” EPIS describes the progression of implementation through 4 key phases. Rather than evaluating outcomes, the model highlights factors inner and outer to agencies that act as barriers or facilitators at each stage.

### 2.5 Data Analysis

Checklist results were aggregated and analyzed at the agency level. Two reviewers (MP and EC) independently coded transcripts for inner and outer factors utilizing a deductive framework-guided approach based on the EPIS framework. Coders met to resolve discrepancies and reach consensus on code definitions. Codes were then reviewed by the full project team and grouped into higher-order themes through consensus, with attention to relevance and potential research team bias. Themes were subsequently shared with the 6 EMS agencies for feedback to increase accuracy. This study was approved by the University of Missouri-St. Louis institutional review board (IRB), and all coding and analysis were conducted using ATLAS.ti version 24.1.^37^

## 3 Results

Eight trainees representing all 6 pilot EMS agencies participated in interviews, including 4 community paramedics and 4 individuals in EMS leadership roles. Agency characteristics are described in Table 1. All participants were white and evenly distributed by gender (4 women and 4 men). Qualitative analysis produced 40 codes, including 21 outer and 19 inner factors. These codes were synthesized into 11 overarching themes: 5 barriers and 6 facilitators (Table 3).

**Table 3:** Barriers and facilitators to prehospital buprenorphine implementation among emergency medical services (EMS) agencies.

EPIS context		Themes
Barriers	Inner	Stigma among paramedics toward people who use drugs
		Staff resistance to additional responsibilities
		Compassion fatigue among paramedics
	Outer	Limited availability of long-term treatment for patients
		Inconsistent linkage to care post-overdose
Facilitators	Inner	Support of protocol adoption and adherence
		Integration with community paramedic programming
	Outer	Prior exposure to harm reduction training
		Foster-and-frame implementation strategy
		Financial investment from the state

Themes based on semistructured interviews with leadership from 6 pilot EMS agencies in Missouri. Interview items and analysis were informed by the exploration, preparation, implementation, sustainment (EPIS) framework.

### 3.2 Readiness Checklist

Agencies reported being prepared to implement prehospital buprenorphine following frame-and-foster training, with 3 scoring 10 of 10 and an average of 9 of 10 (Table 2). Among those not fully ready, 2 lacked paramedic training plans, citing limited time for professional development and staffing uncertainty; 1 needed stronger peer-referral partnerships, and another had limited medical director support and planned a limited launch. All agencies completed required education, established systems for patient screening and tracking, developed plans to adhere to state and federal regulations for controlled substances, and secured funding for medication.

### 3.3 Inner Agency Barriers

The internal barriers identified within EMS agencies focused on staff willingness and ability to carry out the new practice. Trainees (N = 8) reported that paramedics at their agencies often hold stigmatized views toward people who use drugs and were concerned it would impact uptake, even after training. *“*You wake the same person up with Narcan, and they say, I’ve never even used drugs. And so, you know, they immediately look at that as a dishonest, lying person that isn’t worth their time instead [of] this entire picture of somebody.” (S1)

Trainees noted that even when paramedics follow the protocol, stigma could impact whether they effectively build rapport at the scene or explain the benefits of the mediation to patients. These stigmatized beliefs are compounded by compassion fatigue and burnout from serving on the frontlines of the overdose epidemic. *“*Our clinicians, it’s not that they don’t care. I think they’re probably burned out and beat down. And some of that burnout is tied to witnessing many repeat overdoses, leading to a feeling of futility.” (J3)

Resistance to new responsibilities emerged as an internal barrier. Trainees described emergency response as fast-paced, where staff rapidly address concerns and transport as necessary, and anything resulting in additional time on scene is resisted. One trainee noted, “Red flags went up … Why are we sitting on the scene extra when we’ve got other calls to run?” (J3). Trainees were concerned that such behavior could lead to paramedics skipping the intervention or assessing withdrawal too quickly and inaccurately.

### 3.4 Outer Agency Barriers

Outer barriers included concerns about access to ongoing treatment options after patients received prehospital buprenorphine. Some trainees reported poor hospital handoffs and concerns about patient care, with one stating, “I think these people are falling through the cracks” (J3). Trainees worried whether patients would be able to access buprenorphine after their dose in the field, citing a lack of low-barrier options. Despite participation from community linkage programs in the training group, concerns persisted about connecting patients to peer recovery programs, including confusion about EMS referrals, limited follow-up, and changing eligibility criteria. Broadly, trainees noted that paramedics rarely learn about patient outcomes unless the patient experiences another overdose, limiting insight into treatment effectiveness.

### 3.5 Inner Agency Facilitators

Trainees reported that codeveloping clear induction protocols was a strong facilitator. The training began with buprenorphine induction protocols developed in New Jersey (Supplementary Appendix 4), which agencies adapted internally and then collaboratively to fit local context and capacity, for example, by altering the timing for additional dosing. Trainees emphasized that successful implementation would depend on the clarity of the final protocol for staff at all levels and leadership oversight of adherence. One trainee noted, “[We’re] changing the treatment modality by changing the protocol. Training to address the biases that we know exist and that we know we’re gonna have is an ongoing thing. But as far as from an operational standpoint, when X happens, do Y. We’re completely comfortable with that kind of implementation.” (J1)

Trainees also cited integration with community paramedicine programming as a facilitator in agencies with developed teams. Districts with community paramedic programs (n = 3) reported more capacity for training and better positioning to develop relationships with community providers for linkage-to-care. They also reported additional on-scene time to engage patients and address early implementation challenges. One noted, “That’s the beauty of the role; you have more flexibility to navigate all these systems” (J2).

### 3.7 Outer Agency Facilitators

External facilitators included support from federal and state-funded programs. Trainees valued pilot funding for initial medication supplies, lockboxes, and related materials and believed it facilitated buy-in internally and externally. The 6 months of structured frame-and-foster training were seen as thorough preparation, with emphasis on the value of peer-to-peer learning with fellow EMS trainees. Materials developed by EMS practitioners were a strength they believed would increase uptake within their agency. They valued that subject matter experts remained accessible after implementation through ongoing community-of-practice meetings. One trainee shared, “When I’m trying to talk to doctors, they just want to hear from other doctors. They don’t want to hear from me, and so medics are the same way. We like hearing from other medics. So I think [bringing in EMS experts] was a really good addition.” (S1)

All participating agencies had previously completed grant-funded first responder training in overdose education and naloxone administration and suggested it helped promote readiness to expand response to include prehospital buprenorphine.

## 4 Limitations

While this study provides guidance to EMS providers seeking to integrate prehospital buprenorphine into routine protocols, several limitations should be noted. First, the sample size is small, which may limit generalizability. However, the eight trainees represent EMS agencies from rural and urban Missouri jurisdictions and serve as early stakeholders in implementing this emerging practice. Second, the participating agencies were not randomly selected; they represent districts with the capacity to participate in the training process. Results might not extend to EMS agencies that are resource-limited. The study team had previously trained some of these agencies in overdose response strategies, which might have influenced selection and readiness. Finally, as this study occurred during the preparation phase, it cannot assess how training efforts influenced patient outcomes.

## 5 Discussion

This study described what we believe to be one of the first statewide efforts to train diverse EMS agencies to adopt prehospital buprenorphine protocols. Using an established implementation science framework, we evaluated the potential efficacy of a 6-month frame-and-foster training strategy and identified barriers and facilitators as agencies prepared to integrate protocols into practice. Following training, agencies reported readiness for implementation and pointed to the benefit of codeveloping agency protocols and regular touchpoints with content experts familiar with the challenges faced by paramedics. The “Overdose Chain of Survival” provided a relevant framework to discuss long-term strategies to reduce overdose risk. Trainees did not report concerns related to several barriers reported in previous literature, such as patient side effects, legal implications, inconsistent implementation, or medication access, suggesting the training approach helped mitigate common challenges.^26^^,^^28^ However, findings revealed concerns about integrating new buprenorphine protocols related to staff stigma and burnout. This is consistent with research documenting stigma among first responder groups toward people who use drugs,^38^ fueled by increasing rates of overdose calls for service.^39^ Of note, trainees did not express concerns about buprenorphine risk compensation and stigma toward MOUD noted in other studies.^26^^,^^28^ This suggests targeted education and framing may reduce intervention-related stigma among EMS providers.^40^, ^41^, ^42^

The primary outer barrier was concern about referrals to treatment and recovery services, affecting uptake by both paramedics and patients. This aligns with other findings highlighting limited follow-up and access to outpatient services,^28^ underscoring the need for ongoing cross-sector community approaches beyond an initial training period. Although the community-of-practice group did include representatives from peer treatment-linkage programs, it did not include representatives from hospitals, pharmacies, or substance use treatment agencies, leaving agencies to navigate these connections independently.

Community paramedicine programming was a key inner agency facilitator toward readiness. Although the protocol was designed to be carried out by any EMS crew, the agencies with active community paramedicine programs noted increased time to dedicate to training and the ability to mitigate concerns about additional time on scene and providing non-emergency transportation. As prehospital buprenorphine remains rare,^25^ more work is needed to understand how training and implementation needs vary across agency staffing models.

EMS remains at the forefront of the ongoing overdose epidemic. Administering prehospital buprenorphine, an evidence-based MOUD, has potential to reduce overdose and drug-related harms. However, integrating new practices presents barriers. While future research on Missouri’s statewide effort will focus on the clinical uptake and outcomes, the present study’s focus on program development and training provides insight on how EMS agencies can be prepared to adopt this emerging and promising intervention.

## Author Contributions

MP: Conceptualization, Methodology, Investigation, Data curation, Formal analysis, Writing – original draft, Writing – review & editing. STS: Project administration, Formal analysis, Writing – original draft, Writing – review & editing. JW: Project administration, Investigation, Writing – original draft, Writing – review & editing. GC: Writing – review & editing. MK: Writing – review & editing. BR: Conceptualization, Writing – review & editing. RPW: Conceptualization, Writing – review & editing, Supervision, Funding acquisition. All authors reviewed and approved the final manuscript.

## Funding and Support

The authors disclosed receipt of the following financial support for the research, authorship, and/or publication of this article: Opioid Settlement Funds. Funder: Missouri Department of Mental Health. (Contract AOC23380020)

## Conflict of Interest

All authors have affirmed they have no conflicts of interest to declare.
