# Supplementary material for: Preparing Emergency Medical Services for Prehospital Buprenorphine: Examining a Train-the-Trainer Approach to Strengthening the Overdose Chain of Survival
Source: J Am Coll Emerg Physicians Open. 2026 Jul 1;7(4):100452. doi: 10.1016/j.acepjo.2026.100452 (PMC13355182; doi:10.1016/j.acepjo.2026.100452)
Supplement: Supplementary Appendix 1-4 [file mmc1.docx]

**Supplementary Appendix 1**

***Overdose Chain of Survival (analogous to the Cardiac Chain of Survival) used to frame implementation of buprenorphine among* emergency medical services (EMS) *agencies.***


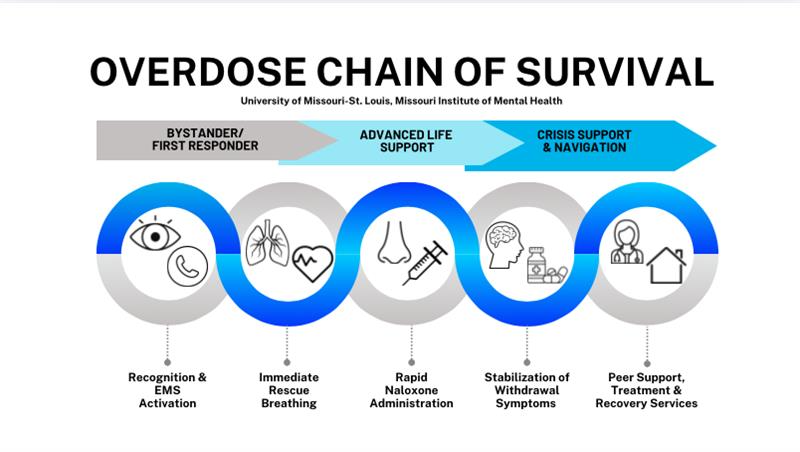


**Supplementary Appendix 2**

***Field Buprenorphine Rescue Challenge Coin***


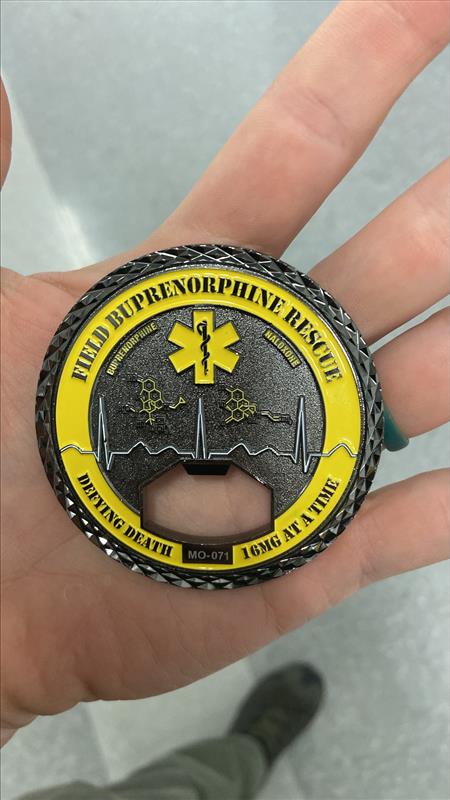


A challenge coin is a metal medallion that typically features an organization's insignia or emblem; they have a long history in military organizations and are used in emergency medical services (EMS) provider agencies to recognize service or achievements.

**Supplementary Appendix 3**

**Readiness checklist and interview guide for assessing EMS agency preparedness for prehospital buprenorphine implementation**

Training & Staffing (Frontline Context)

1. Has a department representative engaged in at least 4 hours of training to learn best practices for implementing the core protocol (e.g. Communities-of-Practice meetings).

1. YES
2. NO
3. I DON’T KNOW

*Prompts:*

- *What areas do you feel most prepared to begin implementation?*
- *What gaps in knowledge do you still have?*

2. Does your department have a plan for how they will train your providers on the prehospital buprenorphine protocol best practices and expectations?

1. YES
2. NO
3. I DON’T KNOW

*Prompts:*

- *How are you planning to conduct training in your department?*
- *When are you hoping to get started?*
- *Do you have any concerns about training your staff?*

3. Does your department have enough paramedics and vehicles to implement this pilot?

1. YES
2. NO
3. I DON’T KNOW

*Prompts:*

- *How many staff are you planning to start piloting this with?*
- *Who are they? How did you choose?*
- *Does your department have a community paramedic program that will be involved in implementing this work and how will they be involved?*

Protocol (Fidelity)
4. Is your department prepared to use the Clinical Opiate Withdrawal Scale (COWS) for evaluating patients post-overdose?

1. YES
2. NO
3. I DON’T KNOW

*Prompts:*

- *What is your department’s current protocol for post-overdose patients?*
- *Do you expect any issues in having paramedics learn and use COWS scoring?*
- *Are you set up to track the COWS score? How?*

5. Is your department prepared to make referrals to Missouri’s Engaging Patients in Care Coordination (EPICC) program or other service linkage programs?

1. YES
2. NO
3. I DON’T KNOW

*Prompts:*

- *If yes, tell us a little bit more about your current relationship with EPICC?*
- *What barriers are you experiencing or expecting to experience connecting patients with EPICC?*

6. Is your department prepared to provide leave-behind naloxone kits following overdose responses?

1. YES
2. NO
3. I DON’T KNOW

*Prompts:*

- *Is that something you currently track or plan to track?*
- *If no, why not?*

7. Does your department have the capacity to track and share administrative data (with appropriate privacy protocols) related to prehospital buprenorphine (such as calls for service)

1. YES
2. NO
3. I DON’T KNOW

*Prompts:*

- *Have you filled out the data sharing agreement? If not, do you expect any barriers to sign off?*
- *Once you get started, what kind of results would indicate success to you?*

District Capacity & Context (Administrative Context)

8. Is your Medical Director willing to support your department’s ongoing participation?

1. YES
2. NO
3. I DON’T KNOW

*Prompts:*

- *How does leadership feel about this initiative? Why?*

9. Buprenorphine is a Schedule III substance. Does your agency have the required state and federal controlled substances registrations?

1. YES
2. NO
3. I DON’T KNOW

*Prompts:*

- *Are there any other policy & regulatory barriers we should know about in carrying and administering bupe?*

10. Does your department have enough financial capacity to purchase medication and other supplies to successfully implement this pilot?

1. YES
2. NO
3. I DON’T KNOW

*Prompts:*

- *Do you have any concerns about the financial component of carrying out this new protocol?*

*Wrap Up:*

- *Do you foresee any barriers to success not listed above?*
- *Are there any resources or support from us, our consultants, or the other pilot districts that would feel helpful right now?*

**Supplementary Appendix 4**

**Core protocol: emergency medical service (EMS) prehospital buprenorphine administration for precipitated withdrawal**

| - The following procedures are authorized for patients who have overdosed on opioids AND received naloxone by EMS provider or bystander:   - Assess patient for any exclusion criteria     - Altered mental status/ no capacity     - Unwilling to give name and DOB     - **Taken any methadone within the past 48 hours -** patients who have taken methadone in the last 48 hours are at risk of severe withdrawal if given buprenorphine     - Patient does not take opiates daily     - Under 18 year olds   ***If any of the above are present, the patient is NOT eligible for buprenorphine, continue with Overdose protocol.     - Calculate a Clinical Opiate Withdrawal Scale (COWS) score (Below)   - Score of less than 7     - The patient is NOT eligible for buprenorphine - Score of 7 or greater   - Counsel patient regarding buprenorphine treatment for withdrawal   - Assess the desire to initiate treatment   - If the patient DECLINES, the patient is NOT eligible for buprenorphine - Patient agreeable to buprenorphine treatment   - 16mg initial SL buprenorphine dose   - 4mg ondansetron SL/IM/IV PRN nausea   - 8mg additional PRN dose of buprenorphine - Give water to moisten mucous membranes   - Administer 16mg SL buprenorphine   - Reassess COWS score after 5-10min     - If improved, proceed to appointment scheduling     - If COWS worsens or not improve, can give an additional 8mg SL buprenorphine - Administer 4mg ondansetron SL/IM PRN nausea - Contact EPICC to help provide post-overdose referrals   - Eastern: 314-819-4275   - Central: 1-800-395-2132   - Western: 1-888-279-8188 |
| --- |
| ** *This protocol is written by Gerard G. Carroll, MD, FAAEM, EMT-P* |
